# Age rationing and prudential lifespan account in Norman Daniels’ *Just health*

**DOI:** 10.1136/jme.2008.024398

**Published:** 2008-12-15

**Authors:** S Brauer

## Abstract

Could age be a valid criterion for rationing? In *Just health,* Norman Daniels argues that under certain circumstances age rationing is prudent, and therefore a morally permissible strategy to tackle the problem of resource scarcity. Crucial to his argument is the distinction between two problem-settings of intergenerational equity: equity among age groups and equity among birth cohorts. While fairness between age groups can involve unequal benefit treatment in different life stages, fairness between birth cohorts implies enjoying approximate equality in benefit ratios. Although both questions of fairness are distinct, the resolution of the one depends on resolution of the other. In this paper, I investigate whether Daniels’ account of age rationing could be defended as a fair way of setting limits to healthcare entitlements. I will focus on two main points. First, I will consider whether the age group problem could be resolved without appealing to a conception of the good. Second, I will demonstrate that the connection between the age group problem and the birth cohort problem runs deeper than Daniels initially thought—and that it ultimately suggests a method for prioritisation in problem solving strategies.

Fiscal scarcity in healthcare systems, even in affluent countries like Switzerland and Germany, calls for setting limits on healthcare entitlements as well as establishing priorities among healthcare services.^i^ Such scarcity of financial resources is expected to grow in the face of demographic aging, and on-going development and implementation of high-cost medical technology, among other factors. Some form of rationing, understood as the distribution of scarce resources within the healthcare system, seems to be unavoidable—and already takes place in clinical practice.^3^ The political question at stake is not *whether* heathcare rationing should be conducted in countries like Switzerland and Germany, but rather, *in which manner* should it be performed.^ii^ Economic models are certainly useful in calculating cost-effectiveness, but can neither set standards for fairness, nor define general healthcare objectives. In order to uphold distributive justice in healthcare, a fair scope and fair criteria of rationing have to be determined by a political deliberative process. Ethical considerations of what inclusion and exclusion criteria for healthcare services are morally permissible have to play a vital role in this process.

Could age be a valid criterion for rationing? In chapter 6 of *Just health: meeting health needs fairly*,^6^ Norman Daniels takes up an earlier approach of his and argues that under certain circumstances, age rationing is prudent, and therefore a morally permissibleiii strategy to tackle problems of resource scarcity. Crucial to his argument is a distinction between two problem-settings of intergenerational equity, namely equity among age groups and equity among birth cohorts. While fairness between age groups can involve unequal benefit treatment in different life stages, fairness between birth cohorts implies enjoying approximate equality in benefit ratios. Although both questions of fairness are distinct, the resolution of the one depends on the resolution of the other. In this paper I will investigate whether Daniels’ account of age rationing could justify a fair way of setting limits to healthcare entitlements. I will focus on two points, one concerning his modified resolution of the age group problem, and the other concerning the interconnectedness of the age group problem and the birth cohort problem.

## NORMAN DANIELS: A CASE FOR AGE RATIONING

Daniels carefully avoids fuelling the suspicion that his account of age rationing amounts to some form of discrimination, namely “ageism”.^8^ He acknowledges that societal aging, namely due to declining fertility rates and longer lifespan, is “maybe the most important public health problem of the 21st century” (p162)^6^ since it profoundly alters the population age structure and profile of healthcare needs. But these empirical changes alone should not be appealed to as *moral* reasons to cut heathcare services for older people, even if they result in high-costs for the healthcare system.^iv^ Justifying age rationing with direct appeal to demographic aging would require either ignoring the political and moral ideal of solidarity existing between adjacent generations as expressed in universal coverage, or fallaciously concluding some normative imperative that based on the fact that the elderly population is growing, their health needs count less. Daniels is far from adopting such an argument. Nevertheless, adjustments to healthcare entitlements will have to be made as the available healthcare budget continues to diminish, and a shrinking labour force persistently contributes to the increasing financial burden of trying to sustain healthcare services. To make these adjustments—as Daniels previously asserts in *Just health care*^9^—it is “prudent” to take age into account. That is, it is a morally permissible strategy to consider age when distributing resources although there is, of course, no prior moral obligation to pursue such a strategy.

Daniels contemplates age rationing in the following manner: first, he rejects the idea of conceptualising different age groups as competing for healthcare resources. According to him, this approach is misleading because it ignores the crucial anthropological fact that we all age. Instead, he suggests that we frame the question about fair distribution of resources between age groups differently—as a problem of a single individual who must reason how to prudently allot healthcare resources across different temporal stages in her life. In other words, Daniels resolves the issue of interpersonal justice by way of intrapersonal reasoning, or intrapersonal transfer of resources and “savings”. This “prudential lifespan account” avoids the charge of ageism by transforming the “problem between us and them” into a “problem about my own life”. All people are treated similarly in a particular stage of life (although birth cohorts reach such a stage at different times). Thus, the requirement of justice in the Aristotelian sense, namely to treat like cases alike and different cases differently, is fulfilled. Or, as Larry Churchill suggests, “So what from a slice-of-life perspective seems unfair appears egalitarian in an over-a-lifetime view”.^10^ ^v^

In fact, the prudential lifespan account does not necessarily lead to age rationing, at least not directly. The fair share of healthcare resources available to the prudent planner has to guarantee a fair share of normal opportunities that she can reasonably expect given her natural skills and talents, and societal circumstances. It is then *her* task to allocate these resources appropriately, so as to carry out a life plan that falls within the limits of a normal opportunity range as defined for a given society. She might come to the conclusion that in light of the effects of resource scarcity, not all her healthcare needs can be met throughout her lifespan; it is best for her to budget resources unequally throughout life in order to make her life “as a whole better than alternatives” (p174).^6^

A decision on how to allocate resources over ones lifespan that is considered prudent by the individual would similarly be considered prudent by participants of a fair deliberative process deciding how to distribute healthcare within a society. Daniels is confident that these participants—whom he considers to be “fair-minded people”—could generalise individual plans and converge on a common social security scheme. Moreover, their agreement would also give justificatory ground for what they agreed upon. If they agreed that unequal provision of healthcare to the young and old would be beneficial to all of society, then age rationing would be justified, or so Daniels argues.

It seems evident that the decision to ration based on age is not determined in advance for every society, since it would depend on the particular economic and political situation of that society. Note that Daniels regards age rationing as essentially a last resort; it is allowed only if a more prudent alternative is not available. Whether age rationing is a prudent option, presumably depends on the wealth of a nation and a lack of rational alternatives. Thus, according to Daniels, pure age-based rationing can be morally permissible if a society faces vast resource scarcity.^vi^

By employing the prudential lifespan account, Daniels defends age rationing as a legitimate rationalisation strategy without making the moral assumption that life at one age is more valuable than at another. He also avoids appealing to duties between the old and the young. Under some circumstances, members of society would just be better off from an age rationing scheme, he argues. Nevertheless, he remains silent on the question of what particular reasons should society give to justify age as a valid criterion for rationing (in addition to, or instead of, other criteria). If Daniels’ prudential lifespan account is in fact a morally permissible, fair and rational way to design a resource allocation scheme, then why not always adopt it when rationing is necessary in the healthcare sector? Considering that even wealthy countries such as Switzerland openly think about rationing policies, a Danielsian form of age rationing could become a prevailing strategy that potentially guided policy decisions.

## QUESTIONS ABOUT THE AGE GROUP PROBLEM

According to Daniels, the moral importance of healthcare stems from the fact that normal species functioning has a significant effect on the opportunity available to an individual. Consequently, there is a societal obligation to guarantee individuals a fair share of the normal opportunity range for their society, given their talents and skills. Insofar as disease and disability impair normal functioning—and thus forestall the enjoyment of a fair share of opportunity range—normal functioning has to be restored (and protected) through healthcare provision. This line of thought prompts the question whether illness related to aging and death would have to be treated at all. Is not aging, as Daniel Callahan^12^ argues, a natural component of life, and thus belongs to “normal species typical functioning”? In line with Christopher Boorse,^13^ Daniels understands disease not as any unwanted condition, but as a departure from normal functioning.vii Therefore, given Daniels’ account, what moral reasons do we have to provide care to a fragile and demented 80-year-old person in the first place, especially if her physical and mental condition could be perceived as resulting from a normal aging process?

Daniels does not explicitly address this question. However, in *Just health* and in his earlier work he mentions an “age-relative normal opportunity range”.^7^ Presumably such an age-relative opportunity range is undermined or impaired in the case of the fragile and demented 80-year-old. If impairment of *fair share of normal opportunities* bears moral weight, rather than the (age-relative or not age-relative) *origin* of impairment, then the provision of care to the 80-year-old is not a matter of compassion or benevolence. It is a matter of justice and fairness to counteract impairment of opportunity for older people. It is important to establish this point in order to understand why Daniels considers the age group problem to be a matter of fairness, and therefore a part of his theory of healthcare justice.

As previously elaborated, Daniels solves the age group problem with the help of a rational thought experiment—the prudential lifespan account. In comparison to his earlier work,^9^ ^15^ he modifies this account in a crucial respect. Initially, he appealed to the concept of “veiled prudence”, a way of deciding under conditions of ignorance in which the planner is blinded to facts about her life and her actual preferences. Veiled prudence would prevent any bias that a “fully conformed rational consumer” might have when asked to allot resources over her lifespan (p51).^15^ In the style of the Rawlsian “veil of ignorance”, under which parties in the “original position” have to decide on principles of justice, Daniels’ veiled prudence consists of four key elements: (1) The prudent planner does not know her age. (2) The prudent planner is ignorant of her conception of the good, although she knows that she might want to alter her conception of the good in the course of her life. Therefore she has an interest in keeping her options open for future revisions. (3) The prudent planner is guided by a time-neutral concern for her wellbeing over the lifespan. (4) The prudent planner has to plan for each stage of her life under the assumption that she will live through it.

These four conditions for prudential planning remerge in chapter 6 of *Just health*. But, compared to what he thought in earlier works, Daniels is more sceptical of whether veiled prudence is enough to guide justice.^7^ In *Just health* he explicitly states: “I also underestimated the sources of disagreement about how to set priorities in meeting needs at different stages of a life by relying on prudence alone. […] we must supplement the [fair equality of (SB)] opportunity principle—even relativised to a stage of life—with fair [deliberative (SB)] process” (p175).^6^ Daniels seems to express the concern that reasonable disagreement between “fair minded people” could emerge in the context of making specific trade-offs when deciding on an age-relative healthcare scheme. Since such disagreement is reasonable, prudence alone cannot lead to a resolution. It has to be accompanied by a “fair deliberative process”, explicated by Daniels as “accountability for reasonableness” (chapter 4).^6^

This alteration of Daniels’ earlier approach is not unexpected. Already in *Am I my parents’ keeper?* Daniels acknowledges that there is no one prudent plan for all individuals (p54),^15^ despite thinking that the lack of information would be sufficient to eliminate differences. However, the planners would have to be informed of societal circumstances in order to decide on a transfer scheme. Such information, including information about demographic aging and evolving patterns of needs could be evaluated differently, depending on an individual’s sensitivity to risk or an individual’s cherishing of the option to revise her conception of the good. There are good reasons to reserve a majority of healthcare resources for the early and middle age during which individuals receive education, work and raise children, and so on. But there are also good reasons to reserve a lot of resources for old age; first, there is less a risk of becoming sick as a young person, and second, due to higher life expectancy, individuals tend to live a lengthy portion of their life in retirement and thus under a restricted financial budget. Achieving an agreement on such reasonable disagreement would, to my mind, signify leaving the dimension of impartial prudence and entering that of policy affairs. This contradicts Daniels’ earlier statement that he intends to reserve the former dimension for the age group problem, and the latter for the birth cohort problem.^7^ This friction in Daniels’ account does not necessarily present a severe problem to his latest theory of justice in healthcare,^6^ the heart of which is “accountability for reasonableness”. Pluralism of reasonable views could well be facilitated in a fair procedural manner of policymaking—and besides arriving to a modus vivendi, a more in depth reasonable agreement might not be required for Daniels.

There is, however, another discrepancy in Daniels’ account that might pose a more serious problem to his approach. In *Just health*, he still appeals to the prudential lifespan account as a way of generating good reasons for age-relative resource allocation. Prudent trade-offs are guided—and justified as fair—when they “make life as a whole better than alternatives” (p174).^6^ Yet, there is likely to be disagreement about what distribution makes life as a whole better. Imagine a person who strongly believes in reincarnation and thus regards her life only as an episode in a series of life forms. Death might not frighten her as much as it does for other people. Her prime goal might be to achieve a higher quality of life, not necessarily to live as many years of her “current life” as possible. She might be ready to trade away total quality of life years in order to have more healthcare resources available in earlier life stagesviii; moreover, she might be willing to risk dying in later life stages because she previously used up resources. For another person, such risk-taking behaviour might be absolutely intolerable. His chief value might be quantity of life years, regardless of the level of quality of life that can be expected. Believing that his life is in the hands of God, it would be morally wrong for him to intentionally risk shortening his lifespan. He might decide to allocate healthcare resources more equally over the course of his life in an attempt to live as long as possible.

These examples highlight the important point that deciding how to budget resources over a lifespan most likely depends on the individual’s conception of a good life, and thus varies according to cultural, historical, religious, political and biographical background. Daniels tries to circumvent this dependency by designing prudent planners who are ignorant of their own conception of the good. However, such blindness cannot be comprehensive because in a deliberative process, prudent planners still have to be able to decide on “what makes life as a whole better”. Even if they were ignorant of their own particular conception of the good, and were just informed about the types of conceptions they could adopt in their society, there still could be disagreement about which conception of the good should be appealed to in deciding “what makes life as a whole better”. Despite Daniels’ intention to design the prudential lifespan account in a purely procedural manner, it might not be a workable account without further work done fleshing out “what makes life as a whole better than alternatives”.

## INTERCONNECTEDNESS OF BIRTH COHORT PROBLEM AND AGE GROUP PROBLEM

Daniels explicitly states that a solution to the age group problem must be compatible with a solution to the birth cohort problem and vice versa, despite both problems being distinct. In the following discussion, I will argue that the interconnectedness of the two problems runs deeper than Daniels initially thought. Contrary to Daniels’ argument,^7^ a ranking of the two types of problems is necessary for reasons of theoretical coherency. Additionally, it is necessary in the face of rapid societal aging and start-up problems of developing countries.

In his earlier work, Daniels carefully asserts that the age group problem has to be “framed” by the “fair equality of opportunity principle” (p47).^15^ ^7^ That is, the opportunity principle does not guide prudent allocation of healthcare resources over the lifespan directly; rather, it defines the conditions under which prudent allocation takes place (p178).^6^ ^7^ In a pointed remark, Daniels explains the meaning of framing: “These principles of justice define the overall budget that prudent deliberators must allocate over the lifespan. […] Put more simply, for prudent budgeting, a budget is needed, but what the budget is must depend on what is just, on what people are entitled to” (pp53, 63).^15^ Daniels makes the assumption that there are limits to fairness (defined by the “fair equality of opportunity principle”), which determine the budget for healthcare services. These limits are not at the disposal of prudent planners. Instead, prudent planners must decide on how to allocate healthcare resources over the lifespan *within* these limits. The task of defining the budget for prudent planners is part of the solution to the birth cohort problem. Consequently, Daniels’ claim that *framing* the issue of prudent allocation over the lifespan by principles of fairness already suggests that solving the birth cohort problem is prior to solving the age group problem—prudent planners cannot budget if they do not have a defined budget available.

A closer look at Daniels’ solution of the birth cohort problem is necessary to understand his explanation for why cohorts should have an interest in fair cooperation and budget distribution among each other. His argument for the motivation to cooperate is built on the assumption that there are two common interests among cohorts: all aspire to solve the age group problem effectively through stable institutions, and all aim to share risks across cohorts. Accordingly, cooperation becomes a matter of prudence. If cohorts cooperate, then they can circumvent uncertainty about population, economic growth rates, and technological change, which subsequently may affect productivity (p182f).^6^ The willingness to share risks is a crucial factor in sustaining the stability of institutions. Conversely, expected stability builds trust in institutions and motivates cohorts to share risk since intercohort transfer schemes are considered to be fair over time. However, what is the consequence of a diminishing common interest in risk sharing? On Daniels’ account, erosion of intercohort solidarity would ultimately result in a loss of the incentive for solving intergenerational problems together. Daniels repeatedly states that the prudential lifespan account, which allows unequal resource distribution over the lifespan under fairness considerations, could only succeed if institutions are trusted as being stable and fair over time (p51)^15^ (pp181–5).^6^ Otherwise, the claim that we all age *equally* in respect to uneven healthcare entitlements granted at different life stages, would no longer be valid. The preconditions of stability and fairness of transfer schemes could be regarded as theoretical constraints within an “ideal theory” in which choosers behind the “veil of ignorance” can expect earlier benefits to continue throughout their lifetime.^16^ However, Francis remains sceptical of the capacity of an ideal theory to solve real world problems, arguing, “It is quite different to bring age rationing into play in a world of people who have already suffered from injustice, than to consider it for people who will live their full lives in a just society”.^16^ Francis is right to point to the complexity of real world problems that might undermine theoretical constraints. Although, an ideal theory still has the important function of channelling ideas and thoughts about issues of social justice. Consider the following argument:

If a cohort knows with certainty that the economical, political, social, or demographical situation of a past or future cohort is considerably worse, then “sharing risk” would take place in a *knowingly* lopsided manner. When grievances are expected, it is safe to assume that motivation to cooperate with affected cohorts may diminish. That is, the willingness to cooperate might only be guaranteed so long as all cohorts operate under uncertainty. However, in the case of vast societal aging, there is certainty of potential grievances. Daniels admits that the “problem goes beyond uncertainty and errors for we are certain about the strains that rapid societal aging will place on existing transfer schemes in developed countries and the obstacle that aging imposes on establishing new schemes in many developing countries” (p182).^6^ Diminishing labour forces and evolving patterns of needs confer high costs on the remaining working population without assuring them that the same standard of healthcare will be received when they age.^ix^ Under the Danielsian framework, budgets of adjacent cohorts would be of different amplitude. Such differences pose an obstacle to finding a fair solution to the age group problem through prudential lifespan allocation. Adjacent birth cohorts would not age equally due to serious budget variations; thus, the initial rationale that “we all age” would loose its persuasive power for the employment of the prudential lifespan account.

The situation in developing countries offers another concrete example of trust being absent in institutions. Recall that Daniels explicitly allows for pure age rationing under conditions of vast resource scarcity; however, his argument is not cogent when taking into account the vastly different circumstances of aging in developing countries compared to industrialised countries. Developing countries are confronted with extreme poverty, lack of healthcare services, enormous burdens of disease, unjust political institutions, and the likelihood of war. Due to devastating political and economical circumstances, the elderly would have fewer “opportunities” than were available in the past. Under such circumstances, it is unlikely that the elderly would agree—or think it is prudent—to allot more resources to the young than to themselves. This objection, initially raised by Engelman and Johnson,^17^ could be refuted by Daniels in a twofold manner. First, the “fair equality of opportunity principle” should not apply directly to age-relative resource allocation. Second, in the prudential lifespan account, prudent planners do not know whether they belong to the old generation or to the young generation. Nevertheless, the problem remains that trust in institutions must first be established before making the prudential lifespan account work. Therefore, solving the problem of equity among birth cohorts should be given priority. Only under the condition of approximate equality in benefit rations could the prudential lifespan account be used to solve the age group problem, thereby justifying age rationing as a fair way of setting limits to healthcare entitlements.

## Footnotes

i After the USA, Switzerland has the second- and Germany the third-most expansive healthcare system worldwide with approximately 11% of gross domestic product spent on healthcare in 2003. Cf^1^ ^2^

ii Cf^4^ (p21) and the report of the Swiss Academy of Medical Sciences.^5^

iii What is prudent is also morally permissible for,^7^ (p276) although not necessarily a sufficient reason for an accordant policy.

iv Georg Marckmann forcefully argues that there is no direct empirical link between age and healthcare costs but rather one between approximation to death and costs. Demographic aging burdens the healthcare system because the non-working population increases disproportionately.^2^ Various policy remedies could approach this issue, for instance raising retirement age or incentives for child bearing.

v In critic of Daniels, McKerlie demands that justice has to say something about synchronic distribution between different ages as well.^11^ I agree with McKerlie that if we are concerned with the real world situation in which healthcare reductions have to be justified, we do this not to ourselves undergoing a prudential lifespan thought experiment, but to other affected people.

vi Cf^6^ (p178ff) “Pure age rationing” means providing differential treatment to persons of different ages not because of different healthcare needs, but simply and only because they belong to different age groups.

vii On Daniels’ concept of disease cf also Jecker.^14^

viii Daniels mentions the possibility of trading away total quality of life years as well (p175 footnote 9).^6^

ix In *Just health* Daniels discusses this problem with the example of Italy and China.

## References

[1] Schoenenberger AW Stuck AE Health care for older persons in Switzerland: a country profile. *International Health Affairs* June 2006;54:986–90 10.1111/j.1532-5415.2006.00746.x 16776797

[2] Marckmann G Alter als Verteilungskriterium in der gesundheitsversorgung. In: Schöne-Seifert B Buyx A Ach J, eds. *Gerecht behandelt?*Paderborn: Mentis Verlag, 2006:163–81

[3] Hurst S Slowther A Forde R Prevalence and determinants of physician bedside rationing: data from europe. J Gen Intern Med 2006;21:1138–43 16836629 10.1111/j.1525-1497.2006.00551.x PMC1831659

[4] Zimmermann-Acklin M Rationierung im ethischen disput. Positionen und argumente, in rationierung und gerechtigkeit im gesundheitswesen. Beiträge zur debatte in der Schweiz Zimmermann-Acklin M Halter H, eds. Basel: EMH, 2007:57–66

[5] Swiss Academy of Medical Sciences (2007). Rationing in the Swiss health-care system: analysis and recommendations.

[6] Daniels N (2008). Just health: meeting health needs fairly.

[7] Daniels N The prudential life-span account of justice across generations. In Daniels, N: *Justice and justification*. Cambridge: Cambridge University Press, 1996:257–83

[8] Brock DW Review: justice, health care, and the elderly. Philos Public Aff 1989;18:297–312 11651943

[9] Daniels N Just health care Cambridge: Cambridge University Press, 1985

[10] Churchill LR Age-rationing in health care: flawed policy, personal virtue. Health Care Anal 2005;13:137–46 16013527 10.1007/s10728-005-4477-9

[11] McKerlie D Justice between the young and the old. Philos Public Aff 2001;30:152–77

[12] Callahan D Setting limits: medical goals in an aging society New York: Simon and Schuster, 1987

[13] Boorse C On the distinction between disease and illness. Philos Public Aff 1975;5:49–68

[14] Jecker NS Towards a theory of age-group justice. J Med Philos 1989;6:655–80 2614285 10.1093/jmp/14.6.655

[15] Daniels N Am I my parents keeper New York/Oxford: Oxford University Press, 1988

[16] Francis LP Age rationing under conditions of injustice, in: *Medicine and social justice. **Essays on the distribution of health care*. In: Rhodes R, Battin MP, Silvers A, eds. Oxford: Oxford University Press, 2002:270–7

[17] Engelman M Johnson S Population aging and international development. Addressing competing claims of distributive justice. Dev World Bioeth 2007;7:8–18 17355327 10.1111/j.1471-8847.2006.00152.x

